# Electron Capture Dissociation, Electron Detachment Dissociation, and Collision-Induced Dissociation of Polyamidoamine (PAMAM) Dendrimer Ions with Amino, Amidoethanol, and Sodium Carboxylate Surface Groups

**DOI:** 10.1016/j.jasms.2008.06.016

**Published:** 2008-09

**Authors:** Malgorzata A. Kaczorowska, Helen J. Cooper

**Affiliations:** School of Biosciences, University of Birmingham, Edgbaston, Birmingham, United Kingdom

## Abstract

Here, we investigate the effect of the structure (generation) and nature of the surface groups of different polyamidoamine (PAMAM) dendrimers on electron-mediated dissociation, either electron capture dissociation (ECD) or electron detachment dissociation (EDD), and compare the fragmentation with that observed in collision-induced dissociation (CID). ECD and EDD of the PAMAM dendrimers resulted in simple mass spectra, which are straightforward to interpret, whereas CID produced complex mass spectra. The results show that electron-mediated dissociation (ECD and EDD) of PAMAM dendrimers does not depend on the nature of the surface group but tends to occur within the innermost generations. CID of the PAMAM dendrimers showed a strong dependence on the nature of the surface group and occurred mostly in the outer generation. The results demonstrate the potential utility of ECD and EDD as a tool for the structural analysis of PAMAM dendrimers.

## Graphical Abstract

Investigation of the effect of the structure and nature of surface groups of different PAMAM dendrimers on electron-mediated dissociation (ECD, EDD) and collision-induced dissociation (CID).

**Figure:**
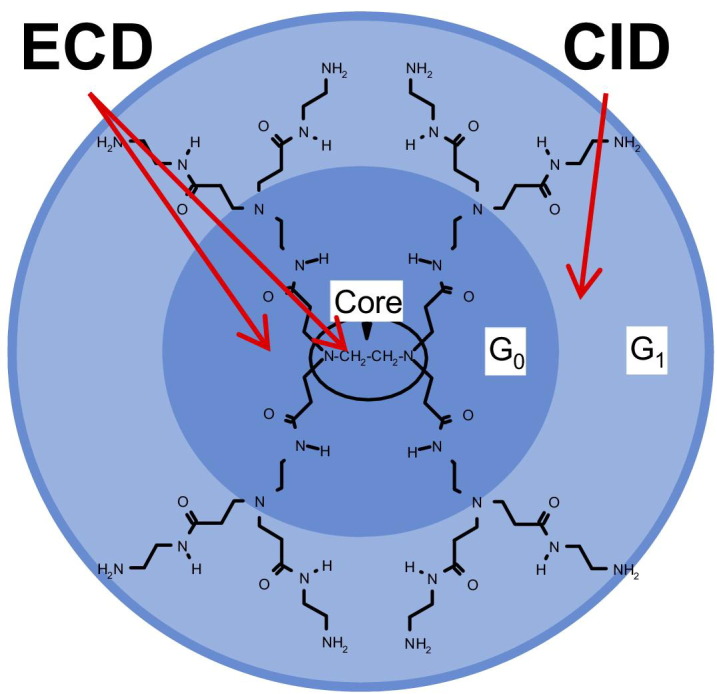

Polyamidoamine (PAMAM) dendrimers, introduced by Tomalia in 1994 [1], are a class of polymers with a high degree of molecular uniformity and a highly functionalized terminal surface. They are characterized by three components: the central core, branches, and surface groups (Scheme 1). Each subsequent growth step represents a new generation of polymer with increasing molecular size, mass, and number of surface groups. Dendrimers allow precise control of size, shape, and placement of functional groups [2, 3], PAMAM dendrimers are often referred to as “artificial proteins” [4] because they closely match the sizes and contours of many proteins. Because of the high level of control over the architecture of dendrimers, these species play important roles as carriers in many biomedical applications including drug delivery, gene transfection, and imaging [4, 5, 6, 7].

**Scheme 1:**
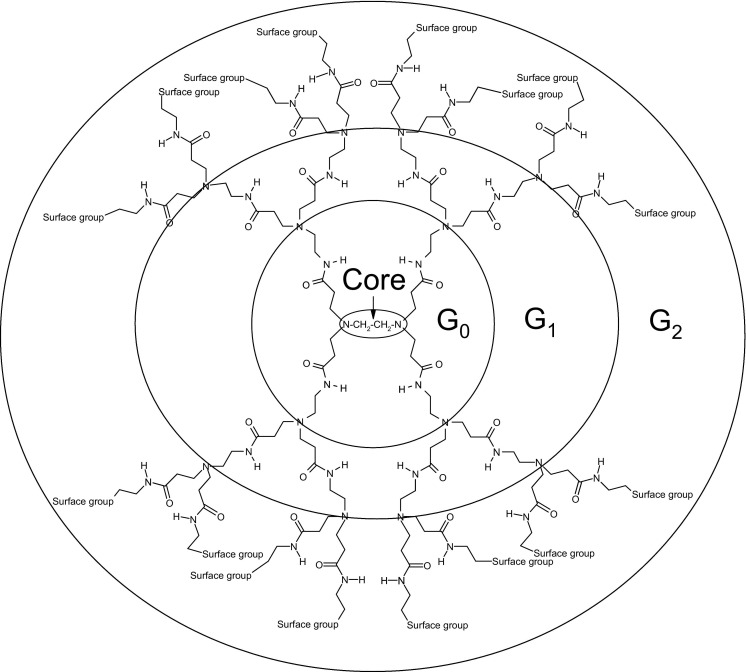
Structure of a PAMAM dendrimer.

As described by Caminade et al. [8], the numerous applications of dendrimers means there is a critical need for techniques for their characterization. Electrospray ionization (ESI) Fourier transform ion cyclotron resonance (FT-ICR) [9] and matrix-assisted laser desorption/ionization (MALDI) time-of-flight (TOF) [10, 11] mass spectrometry have been applied to molecular weight determination of PAMAM dendrimers. Structural deviations in PAMAM dendrimers have been investigated by use of MALDI-TOF mass spectrometry [12, 13], as has their fragmentation [13, 14]. The collision-induced dissociation (CID) of electrosprayed PAMAM dendrimers and their Ag^+^ complexes has been investigated by Brodbelt and colleagues [15]. The CID of anionic PAMAM dendrimers was studied by McLuckey et al. [16]. Oh and colleagues [17] reported the ECD [18] of a third-generation PAMAM dendrimer ions with amidoethanol surface groups. Despite containing amide functionalities, the third-generation PAMAM dendrimer showed ECD fragmentation different from that observed for peptides/proteins. The c/z^**•**^**-**type cleavage, which dominates ECD of peptides and proteins [18], was observed only as a minor process in ECD. The major fragmentation pathway involved cleavage adjacent to the tertiary amine. Pronounced cleavage of the amide bond was also reported. No cleavage of the ethylene bonds was observed. Unlike proteins, PAMAM dendrimers do not contain flexible basic side chains but basic tertiary amines located along the skeleton of dendritic branches. Amide bond cleavage was explained on the basis of intermolecular charge-solvation between the protonated quaternary amine and the amide nitrogen. It was also suggested that the macromolecular properties of the PAMAM molecule may influence ECD fragmentation channels.

Here, we investigate the effect of the structure (generation) and nature of the surface groups of different PAMAM dendrimers on electron-mediated dissociation (either ECD or electron detachment dissociation (EDD)) [19] and compare the fragmentation with that observed in CID. It might be expected that the different properties afforded by different surface groups, such as proton and metal ion-binding affinities [20], could influence the fragments observed. The results suggest that electron-mediated dissociation of PAMAM dendrimers does not depend on the nature of surface groups, but is related to the structure of the polymer: irrespective of surface group, the dominant ECD fragmentation channel for these species is cleavage at the tertiary amine and pronounced amide bond cleavage. The majority of ECD fragmentation occurred within the innermost generations. In contrast to ECD, collision-induced dissociation of the PAMAM dendrimers depended strongly on the nature of surface groups.

## Experimental

### Sample Preparation

Polyamidoamine (PAMAM) dendrimers were purchased from Sigma–Aldrich (Poole, Dorset, UK) and used without further purification. The PAMAM dendrimers with amino and amidoethanol surface groups were diluted to a concentration of 10 pmol/μL in solutions of methanol (FisherScientific, Loughborough, UK) to water (J. T. Baker, Middlesex, UK) to acetic acid (FisherScientific) (49:49:2, vol/vol). The 0.5-generation PAMAM dendrimer with sodium carboxylate surface groups was prepared (10 pmol/μL) in methanol:acetic acid (98:2) solution.

### Mass Spectrometry

Mass spectrometry analysis was performed on a Thermo Finnigan LTQ FT mass spectrometer (Thermo Fisher Scientific, Bremen, Germany). Samples were injected by use of an Advion Biosciences Triversa Nanomate electrospray source (Advion Biosciences, Ithaca, NY, USA). Data acquisition and analysis were conducted using the Xcalibur 2.0 (Thermo Fisher Scientific) software. Mass spectra were acquired at a resolution of 100,000 at *m/z* 400. Precursor ions were selected and isolated for ECD in the linear ion trap before transfer to the ICR cell. Electrons were generated on the surface of an indirectly heated barium tungsten cylindrical dispenser cathode (5.1 mm diameter; Heat Wave Labs, Inc., Watsonville, CA, USA), situated 154 mm from the cell, 1 mm off-axis. The current across the electrode was about 1.1 A. Ions were irradiated with electrons for 70 ms. Each ECD scan consisted of five coadded microscans. CID experiments were performed in the front-end linear ion trap and the fragments transferred to the ICR cell for detection. Precursor ions were selected and isolated in the linear ion trap. CID experiments were performed with helium gas at a normalized collision energy of 35%. Each CID scan consisted of five coadded microscans. All tandem mass spectrometry (MS/MS) spectra were averaged over 30 scans and analyzed manually.

## Results and Discussion

### ECD of PAMAM Dendrimers

#### Assignment of ECD fragments ions of PAMAM dendrimers

**Scheme 2:**
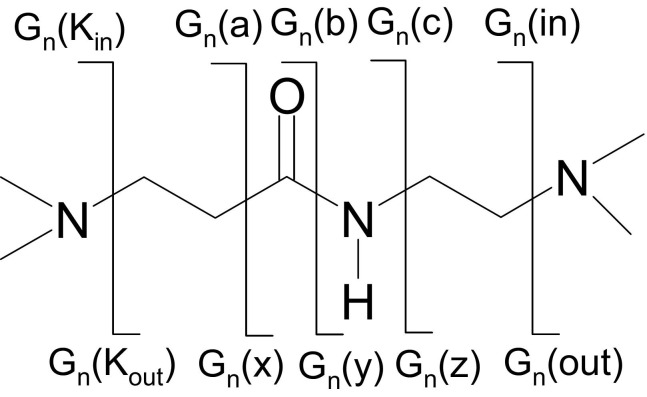
Notations for representing ECD cleavage sites along the backbone of PAMAM dendrimers.

ECD fragments were assigned based on the nomenclature devised by Oh and colleagues [17] (see Scheme 2). Assignments are given in the form G_*n*_(*m*), where subscript *n* refers to the generation in which fragmentation takes place and *m* describes the type of fragmentation; *a*^**•**^/*x*, *b*/*y*, *c*/*z*^*•*^. For example, fragment G_1_(*y*) was derived from cleavage of the amide bond in generation 1. G_*n*_(in) and G_*n*_(out) notations concern fragmentation that takes place core-side of the tertiary amines. K refers to cleavages surface-side of the tertiary amines.

#### Generation 2 PAMAM dendrimer with amidoethanol surface groups (PAMAMG2OH)

The second-generation PAMAM dendrimer with amidoethanol surface groups and ethylenediamine core is a symmetrical molecule that contains three tertiary amine branches (generations 0, 1, and 2) and 16 neutral alcohol surface groups. Electrospray ionization of this PAMAM dendrimer leads to the formation of multiply protonated molecular ions [M + 4H]^4+^ through [M + 7H]^7+^. The most abundant multiply protonated molecular ions of the PAMAM dendrimer—[M + 4H]^4+^, [M + 5H]^5+^, and [M + 6H]^6+^ ions—were isolated and subjected to ECD. Similar fragmentation behavior was observed for each precursor.

**Figure 1:**
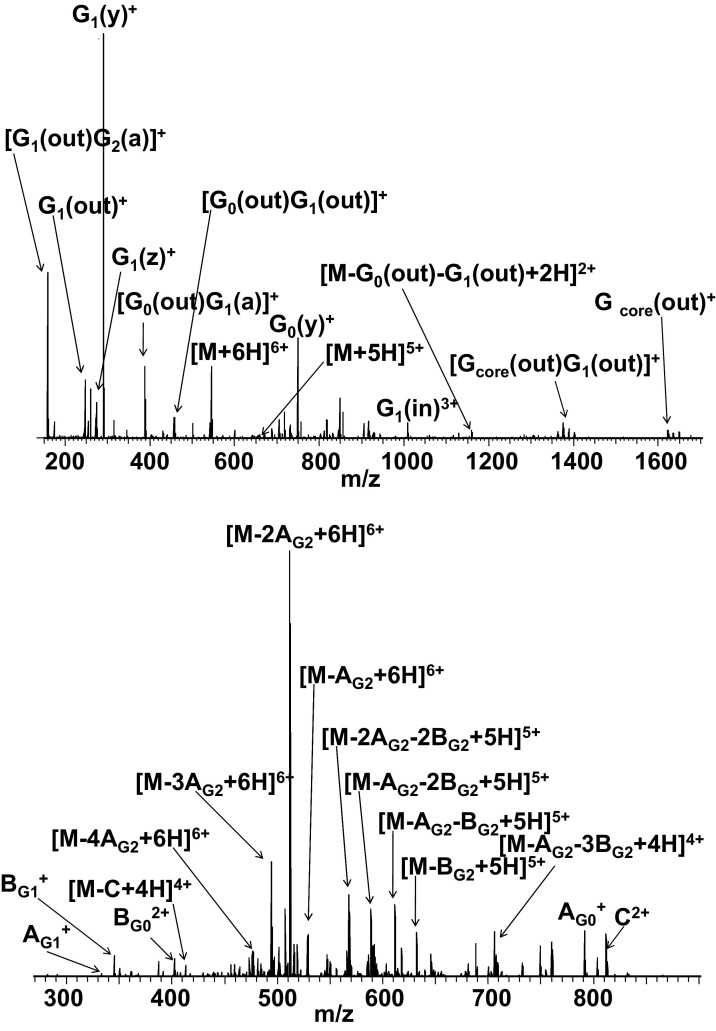
ECD FT-ICR mass spectrum (top) and CID FT-ICR mass spectrum (bottom) of [M + 6H]^6+^ ions of PAMAMG2OH.

The ECD MS/MS spectrum of [M + 6H]^6+^ precursor ions is shown in Figure 1 (top). All fragments detected are described in Supplementary Table S-1, which can be found in the electronic version of this article. The most abundant peaks correspond to *y* fragments from generation 1 and generation zero. No b^(**•**)^ ions were observed. The mass spectrum shows peaks corresponding to triply charged fragments G_1_(in)^3+^, G_0_(in)^3+^ singly charged G_core_(in)^+^, and quadruply charged G_1_(in)^4+^. These four fragments result from cleavage at tertiary amines. In addition, G(out) fragments, which also result from cleavage at tertiary amines but with the charge retained toward the surface of the dendrimer, were observed. These fragments also undergo secondary fragmentations, resulting in singly charged fragment ions [G_1_(out)G_2_(a)]^+^ at *m/z* 160.1205, [G_0_(out)G_1_(a)]^+^ at *m/z* 389.2624, and [G_0_(out)G_1_(out)]^+^ at *m/z* 459.2915. The c/z^**•**^ cleavages that are the most abundant in the ECD of peptides/proteins were found here only as minor channels: G_1_(z)^+^ at *m/z* 274.1757 and G_0_(z)^+^ at *m/z* 732.4592.

As mentioned earlier, similar fragmentation patterns were seen for each charge state of PAMAMG2OH. In each case, fragmentation within these cations occurred in the innermost generations, i.e., the core, G_0_ or G_1_. No fragmentation in the outer generation (G_2_) was observed.

#### Generation 1 PAMAM dendrimer with amino surface groups (PAMAMG1NH_2_)

**Figure 2:**
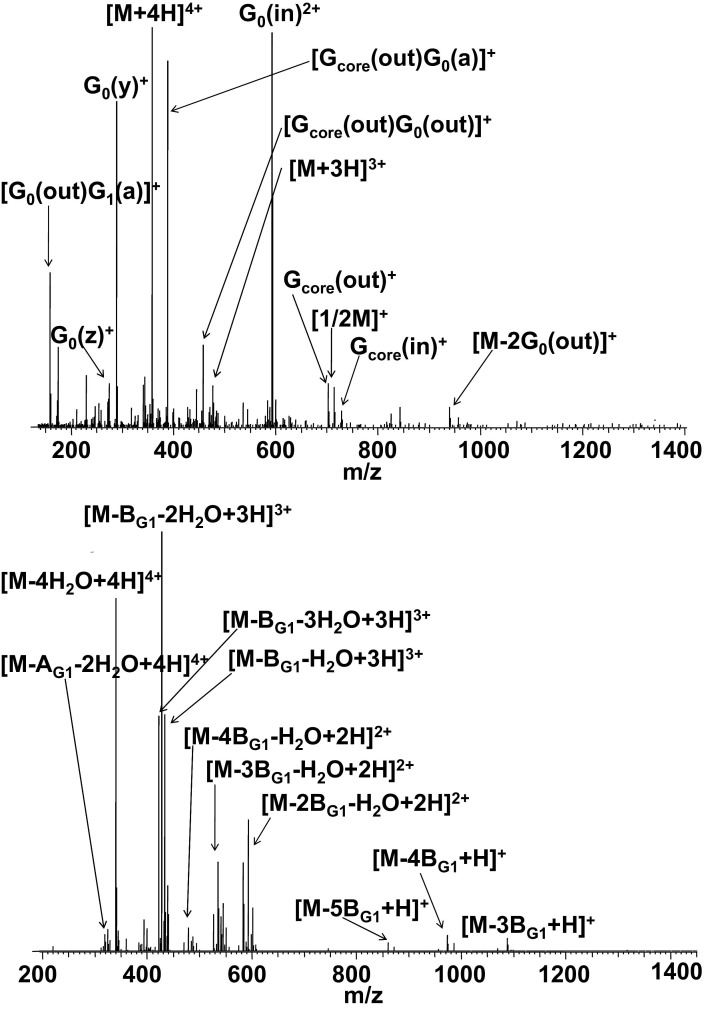
ECD FT-ICR mass spectrum (top) and CID FT-ICR mass spectrum (bottom) of [M + 4H]^4+^ ions of PAMAMG1NH_2_.

Electrospray ionization of the generation 1 PAMAM dendrimer, containing ethylenediamine core, two tertiary amine branches (generations 0 and 1), and eight primary amino surface groups, results in the formation of multiply protonated molecular ions [M + 2H]^2+^ through [M + 4H]^4+^. The fragmentation for each charge state was very similar. Mass spectra shown here were obtained from the most abundant charge state, i.e., [M + 4H]^4+^ ions. The ECD mass spectrum of the [M + 4H]^4+^ ions of PAMAMG1NH_2_ is shown in Figure 2 (top) and the fragments observed are detailed in Supplementary Table S-2. The dominant fragments originate from the inner generation (G_0_). The peaks at *m/z* 592.9225 and 289.2342 can be assigned to G_0_(in)^2+^ and G_0_(y)^+^ fragments, respectively. The c/z^**•**^ cleavages are found only as a minor channel. Other abundant fragment ions at *m/z* 388.3021 and 159.1366 are attributed to secondary fragmentation and can be assigned to [G_core_(out)G_0_(a)]^+^ and [G_0_(out)G_1_(a)]^+^, respectively. These fragments are presumably the result of cleavage of the amide bond followed by loss of CO, together with cleavage at the tertiary amine. Secondary fragmentation reactions involving *a* cleavages were seen for both PAMAMG1NH_2_ and PAMAMG2OH.

Comparison of the ECD mass spectra obtained from PAMAMG1NH_2_ and PAMAMG2OH dendrimers leads to the conclusion that, despite the different chemical properties, the nature of the surface groups does not affect the ECD fragmentation behavior of these polymers. In both cases the mechanism of ECD is bound up with protonation of the tertiary amines and the presence of amide functionalities in the polymer backbone and can be explained on the basis of a charge-solvation model (proton sharing) [17]. The generation number has a more significant influence on the electron capture dissociation. In both cases the ECD mass spectra are dominated by fragments that come from the inner generation(s). The most abundant fragments are of the same type for each dendrimer; G_*n*_(y), G_*n*_(in) and G_*n*_(out), where *n* stands for the inner generation(s) (*n* = 0, 1, in the case of PAMAMG2OH; *n* = 0 in the case of PAMAMG1NH_2_).

### CID of PAMAM Dendrimers

#### Assignment of CID fragments ions of PAMAM dendrimers

**Scheme 3:**
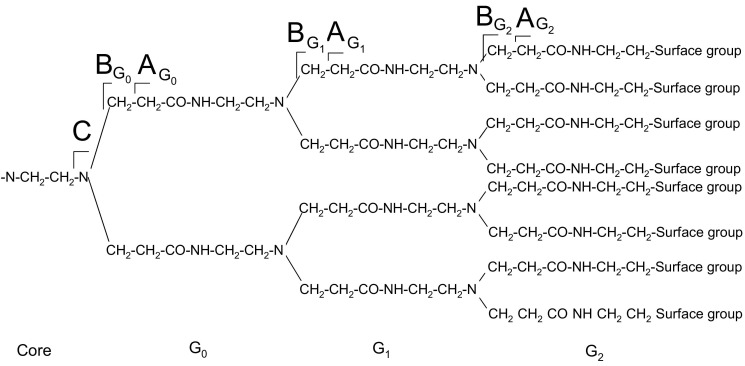
Notations for representing the CID fragmentation sites along the backbone of PAMAM dendrimers (only half of the molecule is shown).

CID fragments were assigned according to the nomenclature shown in Scheme 3. Assignments are given in form **A**_**G***n*_, **B**_**G***n*_, and **C**_**G***n*_, where subscript G*n* refers to the generation in which cleavage takes place. Note that **B**_**G***n*_ is equivalent to ECD assignment G_*n*_(Kout), but the multiplicity of the CID fragmentation (see following text) demands simpler nomenclature.

#### Generation 2 PAMAM dendrimer with amidoethanol surface groups (PAMAMG2OH)

The collision-induced dissociation of [M + 6H]^6+^ ions of PAMAMG2OH (Figure 1, bottom), is dominated by the loss of two neutral fragments, **A_G2_** (Δm = 103.0633) and **B_G2_** (Δm = 115.0633) and combined losses of the two in various stoichiometries. All fragments observed are detailed in Supplementary Table S-3. There are two possible isomers for fragment **A_G2_**, CH_3_—C(O)—N(H)—CH_2_—CH_2_—OH and CH_2_=C(OH)—N(H)—CH_2_—CH_2_—OH. The precise connectivity of this fragment is unknown: The proton localized on the tertiary amine can be bound to the oxygen atom from carbonyl group. As a result of proton transfer and rearrangement, the fragment CH_2_=C(OH)—N(H)—CH_2_—CH_2_—OH may be formed. Neutral fragment **B_G2_** is the result of cleavage at the tertiary amine in the second generation and has the formula CH_2_=CH—C(O)—N(H)——CH_2_—CH_2_—OH. Other minor fragmentation pathways were also found (see Scheme 3).

Fragment ions are observed following CID that result from loss of the same neutral, but differ by charge. For example, the most abundant peak (*m/z* 511.6547) can be assigned to the loss of two **A_G2_** neutrals; i.e., [M − 2A_G2_ + 6H]^6+^ and the peak at *m/z* 613.7838 can be assigned to [M − 2A_G2_ + 5H]^5+^. In general, we observe series of peaks that can be assigned to combined losses of various stoichiometries of **A_G2_** and **B_G2_** for charge states +6 through +3. For example, for the +5 charge state, the following ions were detected : [M − A_G2_ + 5H]^5+^, [M − B_G2_ + 5H]^5+^, [M − 2A_G2_ + 5H]^5+^, [M − A_G2_ − B_G2_ + 5H]^5+^, [M − 2B_G2_ + 5H]^5+^, [M − 3B_G2_ + 5H]^5+^, [M − 2A_G2_ − B_G2_ + 5H]^5+^, [M − A_G2_ − 2B_G2_ + 5H]^5+^, [M − 4A_G2_ + 5H]^5+^, [M − 3A_G2_ − B_G2_ + 5H]^5+^, [M − 2A_G2_ − 2B_G2_ + 5H]^5+^, and [M − A_G2_ −3B_G2_ + 5H]^5+^. In all dissociation reactions, hydrogen migration followed by cleavage at tertiary amine (fragments **B_G2_**, **B_G1_**, **B_G0_**) or between two carbon atoms (fragments **A_G2_**, **A_G1_**, **A_G0_**) leads to losses of amidoamine branches from different generations. Comparison of our results for [M + 6H]^6+^ ions with those obtained for both singly and doubly charged precursors by Brodbelt et al. [15] suggests that CID of PAMAM dendrimers does not depend on the number of mobile protons present.

#### Generation 1 PAMAM dendrimer with amino surface groups (PAMAMG1NH_2_)

**Scheme 4:**
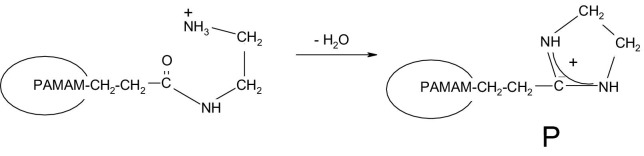
Proposed mechanism for loss of water from PAMAMG1NH_2_.

The collision-induced dissociation of [M + 4H]^4+^ ions of PAMAMG1NH_2_ (Figure 2, bottom), is dominated by loss of water; loss of two neutral fragments **A_G1_** [CH_2_=C(OH)—N(H)—CH_2_—CH_2_—NH_2_, Δm = 102.0793, equivalent to **A_G2_** in PAMAMG2OH] and **B_G1_** [CH_2_=CH—C(O)—N(H)—CH_2_—CH_2_—NH_2_, Δm = 114.0793, equivalent to **B_G2_** in PAMAMG2OH]; and combined losses of water and **A_G1_** and **B_G1_** in various stoichiometries (see Supplementary Table S-4). Such fragmentation behavior is quite surprising, particularly with regard to the CID of PAMAMG2OH, in which no loss of water was observed. We speculate that the unusual CID behavior of PAMAMG1NH_2_ dendrimer proceeds as shown in Scheme 4. The loss of water is pronounced probably because **P** is particularly stable as a result of charge delocalization between the two nitrogen atoms. This idea is supported by a recent study of deamination and dehydration processes of N-terminal glutamine in CID of protonated peptides [21]. Deamination and dehydration processes strongly depend on the presence of “mobile protons.” When mobile protons are present the predominant neutral loss process from N-terminal glutamine is elimination of water because of formation of a protonated five-member aminopyrroline ring. When no mobile protons are present, deamination is observed as a result of formation of a neutral pyrrolidinone ring. Both reactions depend on the charge state and stability. In the present case of quapruply protonated PAMAMG1NH_2_ ions, four protons are present and a maximum of four molecules of water are eliminated during CID. In contrast, CID of both singly and doubly charged PAMAMG1NH_2_ ions resulted in loss of ammonia but not loss of water [15]. Neutral losses observed in the CID of PAMAMG1NH_2_ depend on the charge state; for higher charge states, the dominant process is loss of water and, for lower charge states, elimination of NH_3_ is observed.

Unlike ECD, the results of the CID experiments performed for PAMAMG2OH and PAMAMG1NH_2_ suggest a strong dependence of the dissociation processes on the nature of the surface groups. For PAMAMG1NH_2_, the observed CID also depends on charge state. For both PAMAMG2OH and PAMAMG1NH_2_, dissociation occurs mainly in the outermost generation.

### EDD and CID of Anionic Polyamidoamine (PAMAM) Dendrimer, Generation 0.5, with Sodium Carboxylate Surface Groups

**Figure 3:**
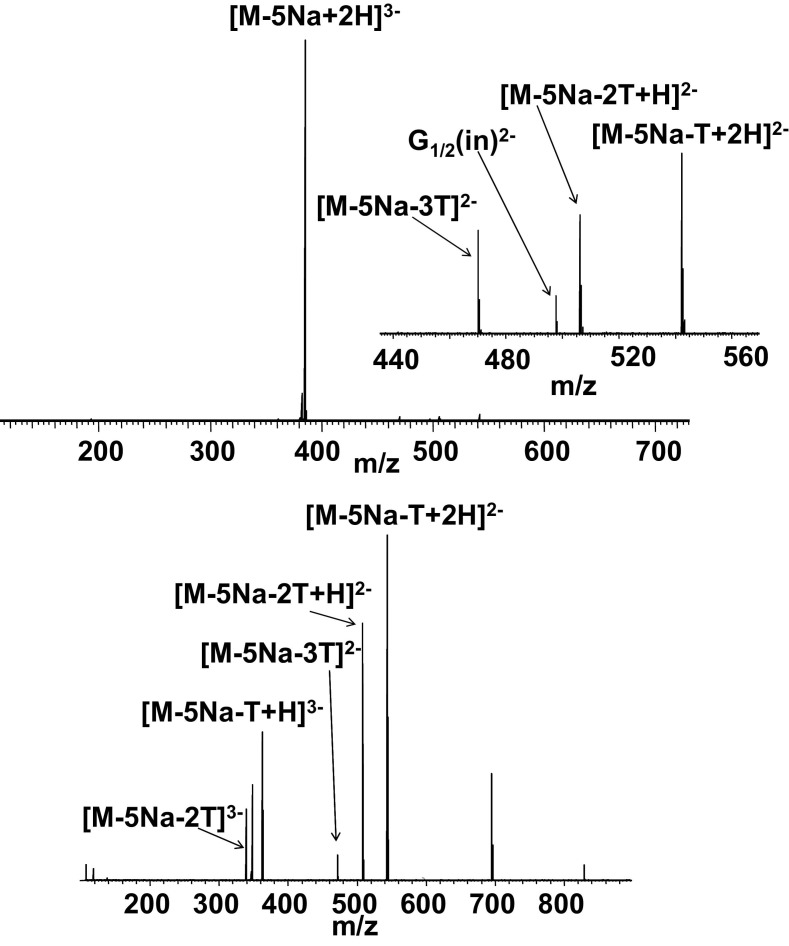
EDD FT-ICR mass spectrum (top) and CID FT-ICR mass spectrum (bottom) of [M − 5Na + 2H]^3−^ ions of PAMAMG0.5COONa.

The PAMAM dendrimer, generation 0.5, with eight sodium carboxylate surface groups and ethylenediamine core (PAMAMG0.5COONa), forms multiply charged anions via negative electrospray. The dendrimer ions have mixtures of sodium and proton counterions because protons present in the MeOH/NH_4_OH solution compete with sodium ions associated with the surface groups [16]. Within each charge state, mixtures of counterions were found according to the formula [M − (*n* + *m*)Na + *m*H]^*n*−^, where *n* is charge state (*n* = 2–4), and *m* is the number of protons replacing sodium in the surface groups. Figure 3 shows the EDD and CID mass spectra obtained from [M −5Na + 2H]^3−^ ions. The EDD mass spectrum (Figure 3, top) is dominated by peaks corresponding to the loss of one, two, and three **T** fragments [CH_2_=CH—C(O)—O^−^] (Δm = 71.01333), which results from cleavage at an outer tertiary amine. Loss of **T** fragments is also observed following CID (Figure 3, bottom). The only peak present in the EDD mass spectrum, but not present in the CID mass spectrum, occurs at *m/z* 497.7054 and can be assigned to the [G_1/2_(in)]^2−^ fragment (*m/z*_calc_ 497.7080). This fragment results from cleavage at the tertiary amine, which was also a major fragmentation channel in the ECD of PAMAM cations. No fragments corresponding to cleavage of the amide bond or c/z^•^-type cleavages were observed following EDD.

## Conclusions

We have investigated the electron-mediated dissociation (ECD and EDD) of three PAMAM dendrimers with the aim of determining the effect of the macromolecular properties (number of generations) and the nature of the surface group. In all cases, fragmentation was dominated by cleavage at the tertiary amines with some amide bond cleavages (ECD). The c/z^**•**^-type dissociation, prevalent in ECD of peptides and proteins, is observed only as a minor channel. The results suggest that ECD (and EDD) are independent of the nature of the surface group, but tend to occur within the innermost generations. In contrast, CID of the PAMAM dendrimers tends to occur in the outermost generation and is strongly dependent on the nature of the surface group. In comparison with CID, ECD produces simple mass spectra that are straightforward to interpret. The results demonstrate the potential utility of ECD as a tool for the structural analysis of PAMAM dendrimers.
