# Supplementary material for: Electron Capture Dissociation, Electron Detachment Dissociation, and Collision-Induced Dissociation of Polyamidoamine (PAMAM) Dendrimer Ions with Amino, Amidoethanol, and Sodium Carboxylate Surface Groups
Source: J Am Soc Mass Spectrom. 2008 Sep;19(9):1312–9. doi: 10.1016/j.jasms.2008.06.016 (PMC2571976; doi:10.1016/j.jasms.2008.06.016)
Supplement: Supplementary file 1 [file mmc1.doc]

**Tables**

**Table 1**.The most abundant fragment ions observed following ECD of [M+6H]^6+^ ions of PAMAMG2OH.

m/z Measured mass Theoretical mass Assignment

160.1205 159.1132 159.1133 [G_1_(out)G_2_(a)]^+^

247.1523 246.1450 246.1454 G_1_(out)^+^

260.1601 259.1528 259.1532 [G_1_(x)G_2_(y)]^+^

273.1916 272.1844 272.1848 [G_1_(y)-H_2_O]^+^

274.1757 273.1684 273.1689 G_1_(z)^+^

291.2022 290.1949 290.1954 G_1_(y)^+^

346.2442 345.2369 345.2138 G_1_(K_out_)^+^

389.2624 388.2551 388.2560 [G_0_(out)G_1_(a)]^+^

459.2915 458.2842 458.2853 [G_0_(out)G_1_(out)]^+^

546.0114 3270.0246 3270.0337 [M+6H]^6+^

655.0132 3270.0295 3270.0337 [M+5H]^5+^

706.4438 705.4365 705.4385 G_0_(out)^+^

718.4434 717.4361 717.4385 [G_0_(x)G_2_(y)]^+^

732.4592 731.4519 731.4541 G_0_(z)^+^

749.4855 748.4782 748.4807 G_0_(y)^+^

756.7244 3022.8684 3022.8804 G_1_(in)^4+^

812.0088 1621.9993 1622.0090 G_core_(out)^2+^

818.0090 1634.0034 1634.0095 [1/2 M+2H]^2+^

848.5533 847.5460 847.5491 [G_core_(out)G_0_(a)]^+^

855.8690 2564.5853 2564.5952 G_0_(in)^3+^

917.5741 916.5669 916.5706 [G_core_(out)G_0_(out)]^+^

926.2467 2775.7182 2775.7273 [M-2G_1_(out)+3H]^3+^

1008.3916 3022.8894 3022.8804 G_1_(in)^3+^

1159.7222 2317.4444 2317.4419 [M-G_0_(out)-G_1_(out)+2H]^2+^

1283.2978 2564.5956 2564.5952 G_0_(in)^2+^

1375.8549 1374.8477 1374.8557 [G_core_(out)G_1_(out)]^+^

1388.8613 2775.7089 2775.7276 [M-2G_1_(out)+2H]^2+^

1623.0055 1621.9982 1622.0090 G_core_ (out)^+^

1635.0068 1633.9995 1634.0090 [1/2 M+H]^+^

1649.0203 1648.0131 1648.0247 G_core_(in)^+^

1860.1514 1859.1441 1859.1567 [M-2G_0_(out)]^+^

**Table 2.** The most abundant fragment ions observed following ECD of [M+4H]^4+^ ions of PAMAMG1NH_2_.

m/z Measured mass Theoretical mass Assignment

159.1366 158.1293 158.1293 [G_0_(out)G_1_(a)]^+^

246.1921 245.1848 245.1852 G_0_(out)^+^

274.2234 273.2161 273.2165 G_0_(z)^+^

289.2342 288.2269 288.2274 G_0_(y)^+^

317.2290 316.2217 316.2223 G_0_(x)^+^

343.2445 342.2372 342.2379 G_0_(K_out_)^+^

349.2566 1392.9972 1392.9991 [M-2H_2_O+4H]^4+^

353.7592 1411.0076 1411.0099 [M-H_2_O+4H]^4+^

358.2617 1429.0176 1429.0204 [M+4H]^4+^

388.3021 387.2948 387.2957 [G_core_(out)G_0_(a)]^+^

457.3231 456.3158 456.3172 [G_core_(out)G_0_(out)]^+^

471.3430 1411.0071 1411.0099 [M-H_2_O+3H]^3+^

477.3460 1429.0380 1429.0204 [M+3H]^3+^

535.3909 1068.7672 1068.7720 [M-G_0_(K_out_)-H_2_O+2H]^2+^

544.3967 1086.7788 1086.7825 G_0_(K_in_)^2+^

579.9147 1157.8148 1157.8196 G_0_(c)^2+^

583.9172 1165.8198 1165.8247 [M-G_0_(out)-H_2_O+2H]^2+^

592.9225 1183.8304 1183.8352 G_0_(in)^2+^

614.4433 613.4360 613.4387 [G_core_(out)G_1_(a)]^+^

702.5060 701.4987 701.5024 G_core_(out)^+^

714.5060 713.4987 713.5024 [1/2M]^+^

728.5214 727.5141 727.5180 G_core_(in)^+^

939.6509 938.6436 938.6501 [M-2G_0_(out)]^+^

**Table 3**. The most abundant fragment ions observed following CID of [M+6H]^6+^ ions of PAMAMG2OH.

m/z Measured mass Theoretical mass Assignment

333.2119 332.2046 332.2060 A_G1_^+^

345.2118 344.2045 344.2060 B_G1_^+^

361.4802 1441.8916 1441.8979 [M-C-2A_G2_+4H]^4+^

387.2458 1544.9540 1544.9613 [M-C-A_G2_+4H]^4+^

402.2509 802.4872 802.4912 B_G0_^2+^

413.0113 1648.0160 1648.0247 [M-C+4H]^4+^

453.2877 2261.4020 2261.4155 [M-2A_G1_-B_G1_+5H]^5+^

455.6879 2273.4030 2273.4155 [M-3A_G1_+5H]^5+^

473.9002 2364.4645 2364.4790 [M-B_G0_-A_G2_+5H]^5+^

476.3002 2376.4645 2376.4790 [M-A_G0_-A_G2_+5H]^5+^

477.3010 2857.7622 2857.7803 [M-4A_G2_+6H]^6+^

494.4780 2960.8242 2960.8438 [M-3A_G2_+6H]^6+^

496.9126 2479.5265 2479.5425 [M-A_G0_+5H]^5+^

511.6547 3063.8844 3063.9070 [M-2A_G2_+6H]^6+^

528.8323 3166.9500 3166.9702 [M-A_G2_+6H]^6+^

541.6734 1621.9983 1622.0090 C^3+^

547.3437 2731.6820 2731.7011 [M-2A_G2_-A_G1_+5H]^5+^

550.3451 1648.0134 1648.0247 [M-C+3H]^3+^

565.3569 2821.7480 2821.7803 [M-A_G2_-3B_G2_+5H]^5+^

567.7602 2833.7645 2833.7803 [M-2A_G2_-2B_G2_+5H]^5+^

570.1592 2845.7595 2845.7803 [M-3A_G2_-B_G2_+5H]^5+^

572.5589 2857.7580 2857.7803 [M-4A_G2_+5H]^5+^

585.9682 2924.8045 2924.8438 [M-3B_G2_+5H]^5+^

588.3725 2936.8260 2936.8438 [M-A_G2_-2B_G2_+5H]^5+^

590.7715 2948.8210 2948.8438 [M-2A_G2_-B_G2_+5H]^5+^

592.1224 2364.4604 2364.4792 [M-A_G2_-B_G0_+4H]^4+^

611.3838 3051.8825 3051.9071 [M-A_G2_-B_G2_+5H]^5+^

613.7838 3063.8825 3063.9071 [M-2A_G2_+5H]^5+^

617.8879 2467.5223 2467.5425 [M-B_G0_+4H]^4+^

631.9963 3154.9450 3154.9704 [M-B_G2_+5H]^5+^

634.3962 3166.9445 3166.9704 [M-A_G2_+5H]^5+^

649.4096 2593.6092 2593.6217 [M-A_G1_-B_G1_+4H]^4+^

652.4061 2605.5952 2605.6217 [M-2A_G1_+4H]^4+^

655.1614 2616.6164 2616.6378 [M-2A_G2_-B_G2_-A_G1_+4H]^4+^

680.9267 2719.6776 2719.7011 [M-A_G2_-B_G2_-A_G1_+4H]^4+^

706.4392 2821.7276 2821.7805 [M-A_G2_-3B_G2_+4H]^4+^

760.4725 1518.9304 1518.9457 [C-A_G2_+2H]^2+^

791.4902 790.4829 790.4912 A_G0_^+^

803.4901 802.4828 802.4912 B_G0_^+^

812.0032 1621.9918 1622.0090 C^2+^

**Table 4**.The most abundant fragment ions observed following CID of [M+4H]^4+^ ions of PAMAMG1NH_2_.

m/z Measured mass Theoretical mass Assignment

319.2338 1272.9060 1272.9094 [M-A_G1_-3H_2_O+4H]^4+^

323.7365 1290.9168 1290.9199 [M-A_G1_-2H_2_O+4H]^4+^

328.2391 1308.9272 1308.9305 [M-A_G1_-H_2_O+4H]^4+^

332.7416 1326.9372 1326.9412 [M-A_G1_+4H]^4+^

340.2509 1356.9744 1356.9781 [M-4H_2_O+4H]^4+^

344.7535 1374.9848 1374.9886 [M-3H_2_O+4H]^4+^

349.2561 1392.9952 1392.9991 [M-2H_2_O+4H]^4+^

387.2827 1158.8262 1158.8301 [M-A_G1_-B_G1_-3H_2_O+3H]^3+^

393.2862 1176.8367 1176.8407 [M-A_G1_-B_G1_-2H_2_O+3H]^3+^

399.2897 1194.8472 1194.8512 [M-A_G1_-B_G1_-H_2_O+3H]^3+^

415.3054 1242.8943 1242.8988 [M-B_G1_-4H_2_O+3H]^3+^

421.3088 1260.9045 1260.9094 [M-B_G1_-3H_2_O+3H]^3+^

427.3121 1278.9144 1278.9199 [M-B_G1_-2H_2_O+3H]^3+^

433.3157 1296.9252 1296.9305 [M-B_G1_-H_2_O+3H]^3+^

439.3193 1314.9360 1314.9412 [M-B_G1_+3H]^3+^

469.3462 936.6778 936.6820 [M-4B_G1_-2H_2_O+2H]^2+^

478.3514 954.6882 954.6926 [M-4B_G1_-H_2_O+2H]^2+^

484.3515 966.6884 966.6925 [M-A_G1_-3B_G1_-H_2_O+2H]^2+^

487.3565 972.6984 972.7031 [M-4B_G1_+2H]^2+^

493.3566 984.6986 984.7031 [M-A_G1_-3B_G1_+2H]^2+^

499.3565 996.6984 996.7031 [M-2A_G1_-2B_G1_+2H]^2+^

526.3853 1050.7560 1050.7614 [M-3B_G1_-2H_2_O+2H]^2+^

532.3852 1062.7558 1062.7614 [M-A_G1_-2B_G1_-2H_2_O+2H]^2+^

535.3904 1068.7662 1068.7720 [M-3B_G1_-H_2_O+2H]^2+^

541.3904 1080.7662 1080.7720 [M-A_G1_-2B_G1_-H_2_O+2H]^2+^

544.3956 1086.7766 1086.7825 [M-3B_G1_+2H]^2+^

547.3902 1092.7658 1092.7720 [M-2A_G1_-B_G1_-H_2_O+2H]^2+^

550.3955 1098.7764 1098.7825 [M-A_G1_-2B_G1_+2H]^2+^

556.3955 1110.7764 1110.7825 [M-2A_G1_-B_G1_+2H]^2+^

583.4241 1164.8336 1164.8407 [M-2B_G1_-2H_2_O+2H]^2+^

589.4241 1176.8336 1176.8407 [M-A_G1_-B_G1_-2H_2_O+2H]^2+^

592.4293 1182.8440 1182.8512 [M-2B_G1_-H_2_O+2H]^2+^

598.4291 1194.8436 1194.8512 [M-A_G1_-B_G1_-H_2_O+2H]^2+^

601.4345 1200.8544 1200.8617 [M-2B_G1_+2H]^2+^

607.4345 1212.8544 1212.8617 [M-A_G1_-B_G1_+2H]^2+^

859.6230 858.6157 858.6239 [M-5B_G1_+H]^+^

871.6236 870.6163 870.6239 [M-A_G1_-4B_G1_+H]^+^

973.6998 972.6925 972.7031 [M-4B_G1_+H]^+^

985.6996 984.6923 984.7031 [M-A_G1_-3B_G1_+H]^+^

1087.7753 1086.7680 1086.7825 [M-3B_G1_+H]^+^
